# Migraine and Stroke: “Vascular” Comorbidity

**DOI:** 10.3389/fneur.2014.00193

**Published:** 2014-10-08

**Authors:** Donata Guidetti, Eugenia Rota, Nicola Morelli, Paolo Immovilli

**Affiliations:** ^1^Neurology Unit, Guglielmo da Saliceto Hospital, Piacenza, Italy

**Keywords:** migraine, stroke, ischemic stroke, hemorrhagic stroke, cerebrovascular disease, vascular risk factors

## Abstract

Several comorbidities are associated to migraine. Recent meta-analyses have consistently demonstrated a relationship between migraine and stroke, which is well-defined for ischemic stroke and migraine with aura (MA), even stronger in females on oral contraceptives or smokers. However, there seems to be no clear-cut association between stroke in migraineurs and the common vascular risk factors, at least in the young adult population. Migraineurs also run an increased risk of hemorrhagic stroke, while the association between migraine and cardiovascular disease remains poorly defined. Another aspect is the relationship between migraine and the presence of silent brain lesions. It has been demonstrated that there is an increased frequency of ischemic lesions in the white matter of migraineurs, especially silent infarcts in the posterior circulation territory in patients with at least 10 attacks per month. Although there is a higher prevalence of patent foramen ovale (PFO) in migraineurs, the relationship between migraine and PFO remains controversial and PFO closure is not a recommended procedure to prevent migraine. As an increased frequency of cervical artery dissections has been observed in migrainous patients, it has been hypothesized that migraine may represent a predisposing factor for cervical artery dissection. There still remains the question as to whether migraine should be considered a true “vascular disease” or if the comorbidity between migraine and cerebrovascular disease may have underlying shared risk factors or pathophysiological mechanisms. Although further studies are required to clarify this issue, current evidence supports a clinical management where MA patients should be screened for other concomitant vascular risk factors and treated accordingly.

## Introduction

Migraine and ischemic stroke (IS) are two highly prevalent diseases. A relevant proportion of the population (up to 21% of women, 6% of men) suffer from migraine attacks and 2/1,000 over 65 year-olds will have an IS (1–3). It has been common knowledge for a long time in clinical practice that some migraine with aura (MA) attacks may mimic cerebrovascular accident symptomatology and that migraine aura may, although rarely, act as an acute precipitant of an IS, the so-called “migrainous infarction.”

The case–control study by the Collaborative Group for the Study of Stroke in Young Women (4) in 1975 was the first to report a twofold risk for stroke in women affected by migraine compared to community controls. Since then, a growing body of evidence has demonstrated the association between migraine, mostly MA, and IS. Moreover, MRI-based studies have demonstrated that migraineurs have an increased frequency of subclinical brain infarcts in the posterior circulation territory and that female migraineurs have an increased risk for white matter lesions (WMLs) (5, 6).

The question remains exactly to what extent there is a generalized vasculopathy in migraine or vascular risk factors, genetic traits, or other pathophysiological mechanisms, including the patent foramen ovale (PFO), may play a role in linking migraine to stroke.

This narrative review aimed at the examination of the current evidence on the relationship between migraine and cerebrovascular disease, mainly IS, focusing on the most intriguing questions arising from this issue in the perspective of the clinical practice.

## Migraine as a Risk Factor for Ischemic Stroke: The Evidence

Three meta-analyses have consistently demonstrated a relationship between these two highly prevalent and disabling conditions, i.e., migraine and IS, where MA emerges as risk factor for stroke (Table 1).

**Table 1:** **Meta-analyses addressing the migraine-stroke association**.

	Stroke type	No. of studies included	Heterogeneity	Migraine (M)	Migraine with aura (MA)	Migraine without aura (MO)
Etminan et al. (7)	Ischemic stroke	14 Total; 14 M, 7 MA, 6 MO	M: *R_i_* = 0.00	RR (95% CI) 2.16 (1.89–2.48)	RR (95% CI) 2.27 (1.61–3.19)	RR (95% CI) 1.83 (1.06–3.15)
			MA *R_i_* = 0.49	
			MO *R_i_* = 0.60	
Schurks et al. (8)	Ischemic stroke	25 Total; 9 M, 8 MA, 8 MO	M: *I*^2^ = 65%	RR (95% CI) 1.73 (1.31–2.29)	RR (95% CI) 2.16 (1.53–3.03)	RR (95% CI) 1.23 (0.9–1.69)
			MA: *I*^2^ = 39%	
			MO: *I*^2^ = 39%	
Spector et al. (9)	Ischemic stroke	21 Total; 12 M, 7 MA, 6 MO	M: *I*^2^ = 63.5%	OR (95% CI) 2.04 (1.72–2.76)	OR (95% CI) 2.25 (1.53–3.33)	OR (95% CI) 1.24 (0.86–2.43)
			MA: *I*^2^ = 55.3%	
			MO: *I*^2^ = 47.1%	
Sacco et al. (10)	Hemorrhagic stroke	8 Total, 8 M, 3 MA, 3 MO	M *I*^2^ = 54.7%	OR (95% CI) 1.48 (1.16–1.88)	OR (95% CI) 1.62 (0.87–3.03)	OR (95% CI) 1.39 (0.74–2.72)
			MA *I*^2^ = 62.1%	
			MO *I*^2^ = 45.3%			

*R_i_ statistic: proportion of the total variance for between study variance: small values (< 0.40) indicate lack of heterogeneity*.

*I^2^ statistic: percentage of total variation among studies due to heterogeneity rather than chance; 25% low heterogeneity, 50% medium, 75% high*.

The meta-analysis carried out by Etminan et al. (7) postulated that there was an increased risk of IS in individuals with migraine: relative risk (RR) 2.16 (95% CI 1.89–2.48), with a higher risk for migraineurs with MA, RR 2.27 (95% CI 1.61–3.19), than those without, RR 1.83 (CI 95% 1.06–3.15), which was even more evident in females taking oral contraceptives, RR 8.72 (95% CI 5.05–15.05).

Another meta-analysis, carried out by Schurks et al. (8), included 25 heterogeneous studies and demonstrated that there was a specific increase in the IS risk correlated to each type of migraine (RR 1.73; 95% CI 1.31–2.29), with the highest values for MA (RR 2.16; 95% CI 1.53–3.03), while the percentages were not statistically significant for individuals without aura (RR 1.23; 95% CI 0.90–1.69). Female migraineurs seem to be at the highest risk (RR 2.08; 95% CI 1.13–3.84), those under 45 years of age (RR 2.65; 95% CI 1.41–4.97), and females on oral contraceptives (RR 7.02; 95% CI 1.51–32.68), in particular, if associated to the smoking habit (RR 9.03; 95% CI 4.22–19.34) (8).

A more recent meta-analysis (9) took into consideration 21 studies, for a total of 622,381 patients (13 case–control studies and 8 cohort studies). They demonstrated a total adjusted odds ratio of stroke in migraineurs vs. non-migraineurs of 2.04 (95% CI 1.72–2.76), of 2.25 for MA vs. non-migraineurs (95% CI 1.53–3.33), compared to 1.24 (95% CI 0.86–2.43) for migraineurs without aura vs. non-migraineurs, for only the female gender it was 2.43 (CI 95% 1.80–3.27). However, when only the studies with a limited bias were considered, the data for all the migraineurs vs. the non-migraineurs produced an odds ratio, which was not statistically significant: 1.24 (95% CI 0.86–1.79) (9).

### Frequency of migraine attacks and stroke risk

Case–control studies suggest that the risk of IS increases along with the increase in frequency of the migraine attacks.

One study on a total of 86 females with MA, aged from 20 to 44 and 214 control subjects, demonstrated that individuals with more than 13 attacks per year have a 10-fold higher risk of stroke (11).

Another study, that compared 386 females with IS, aged from 15 to 49, to 614 control subjects without migraine, showed that females who had more than 12 attacks per year with aura probably run a higher IS risk (OR = 1.7; 95% CI 1.1–2.8), while those with fewer attacks were not at higher risk (12).

The data collected in the Women’s Health study (5,130 migrainous women) confirmed a higher risk ratio for female migraineurs with at least 1 attack with aura per week, RR 4.25 (CI 95% 1.36–13.29), compared to those that have fewer attacks, RR 1.90 (95% CI 1.18–3.08) (13).

## Migrainous Infarction

Migraine with aura may be a direct cause of migrainous infarction (14) and the classification made by the International Headache Society (IHS) in 2013 (15) defines it as “*One or more migrainous aura symptoms associated with an ischaemic brain lesion in appropriate territory demonstrated by neuroimaging*.” These lesions occur during the course of a typical migraine attack with aura, in a subject that has had previous attacks, with the difference that the symptoms persist for >60 min.

The diagnostic criteria of migrainous infarction, recently revised by the International Classification of Headache Disorders, third edition (ICHD-III) (beta version, 2013) (15):

- A migraine attack fulfilling criteria B and C.
- Occurring in a patient with MA (cod.1.2) and typical of previous attacks except that one or more aura symptoms persists for >60 min.
- Neuroimaging demonstrates ischemic infarction in a relevant area.
- Not better accounted for by another diagnosis.

Large clinical series (14, 16) reported that the prevalence of migrainous infarction varies between 0.5 and 1.5% of all IS, ranging up to 10–14% of IS in young patients and shows a prevalent involvement of the posterior circulation territory, occurring mostly in young females with MA.

## Subclinical Brain Lesions

### White matter lesions

A cross-sectional study on a population-based sample of Dutch adults aged 30–60, aimed at comparing the prevalence of brain infarcts and WMLs in migraine cases and controls, showed an increased risk for high WML load only in women with migraine; this risk increased along with an increase in attack frequency, but was similar in patients with migraine with or without aura (5).

The population-based, case–control MRI Dutch study CAMERA (6), enrolled 295 migraineurs and 140 controls, aged 20–60, who were interviewed, given a physical examination and a brain MRI scan. There was an increased independent risk of clinically silent WMLs for female migraineurs (OR = 2.0; 95% CI 1.0–4.1). The risk for WMLs was higher in those with more frequent attacks (≥1 attack/month, OR = 2.6; 95% CI 1.2–6.0), whatever the migraine subtype and the effects of cardiovascular risk factors. There were also an increased proportion of brainstem hyperintense lesions (4.4 vs. 0.7%, *p* = 0.04) (6).

The vascular aging (EVA) study (17) on 116 migraine patients, 617 non-headache controls and 47 non-migraine headache controls, reported an overall association of severe headache with increasing volume of WMLs (41% of patients and 31% of controls had a high WML load). This relationship was not specific to migraine alone, but extended to non-migraine headaches (17).

A recent systematic review and meta-analysis showed an increased risk for WMLs only in MA patients (OR = 1.68; 95% CI 1.07–2.65), while no association was found for migraine without aura (MO) patients (18).

Although WMLs have been associated with an increased risk of cognitive decline, results from longitudinal studies show that patients suffering from migraine (both MA and MO) are not at increased risk of cognitive decline. The lack of association between migraine and cognitive decline was not affected by gender, education, baseline cognitive status, APOE or MTHFT genotype, or medication use. Despite the associations observed between migraine and structural brain lesions in some studies, strong evidence of worse cognitive performance among migraineurs with high-structural brain lesion load is lacking (17, 19).

### Silent infarct-like lesions

Silent infarct-like lesions (ILLs) are MRI or CT signal abnormalities defined as non-mass brain parenchymal defects with the same intensity as cerebrospinal fluid and without symptoms. The distinction between cavitated infarction and dilated perivascular spaces may be challenging and was mainly based on the diameter >3 mm. However, although helpful, the threshold of 3 mm is arbitrary and perivascular spaces of >3 mm are not a rare finding (20).

The CAMERA study (6) reported an increased prevalence of ILLs in the posterior circulation territory in migraineurs. The majority (88%) of infratentorial ILLs had a vascular border zone location in the cerebellum, their prevalence was different between controls (0.7%), patients with MO (2.2%), and patients with MA (7.5%) (OR = 13.7; 95% CI 1.7–112).

Overall, silent ILLs were reported as being more common in migraineurs than in controls in the recent, aforementioned meta-analysis (18). The association of ILLs was greater for MA than for MO, but no association was found for MA (*p* = 0.52) and MO (*p* = 0.08) compared to controls (18).

The progression of ILLs was determined by the AGES-Reykjavik study (21), which evaluated the presence of ischemic lesions in a total of 4,689 individuals, followed as from 1967, examined and interviewed about migraine features and cardiovascular risk factors in midlife. They were given a brain MRI assessment between 2002 and 2006, after an average of 25 years from first examination, 12% of the participants had migraine. After adjusting for other risk factors, MA had an increased RR for ischemic lesions at an advanced age (adjusted OR = 1.4; 95% CI 1.1–1.8), there was an association between MA and ischemic brain lesions in females, with an adjusted OR of 1.9 (95% CI 1.4–2.6). Subjects with MA had a 12-fold risk of ischemic WMLs than controls. There was no increased risk in MO or non-migraine headache (21).

## Migraine as a Risk Factor for Hemorrhagic Stroke: The Evidence

The association between migraine and MA status with the risk of hemorrhagic stroke (HS) in women was assessed in a prospective cohort study, analyzing data from participants in the Women’s Health Study (22). A total of 27,860 women over 45 were studied, 5,130 were migraineurs and 40% had MA. A total of 85 HSs were observed during the 13.6-year-follow-up, with no statistically significant overall difference between subjects with or without migraine. However, there was a statistically significant increased risk for females with active MA (OR = 2.25; 95% CI 1.11–4.54), who had suffered attacks in the previous year. Four HSs per year per 10,000 females are attributable to migraine, while migrainous females without aura have no increased risk of HS (22).

Also, a population-based longitudinal cohort study from Taiwan supports an increased risk for HS in migraineurs. The study enrolled 20,925 subjects with a diagnosis of migraine and 104,625 age- and sex-matched, randomly sampled migraine-free subjects. During the 2-year-follow-up, 113 subjects (0.54%) in the migraine group and 255 (0.24%) in the non-migraine group, developed HS. The crude hazard ratio (HR) for developing HS in the migraine group was 2.22 compared to the non-migraine group (95% CI 1.78–2.77, *p* = 0.0001) and the adjusted HR was 2.13 (95% CI 1.71–2.67; *p* = 0.0001) after controlling for demographic characteristics and comorbid medical disorders (23).

A recent meta-analysis (10) of 8 studies (4 case–control and 4 cohort studies), involving a total of 1,600 HS, showed that the overall pooled adjusted effect estimate of HS in subjects with any migraine vs. control subjects was 1.48 (95% CI 1.16–1.88; *p* = 0.002). The 4 case–control studies had a pooled OR of HS of 1.41 (95% CI 1.09–1.82; *p* = 0.009), whereas the 4 cohort studies had a pooled HR of 1.47 (95% CI 0.97–2.24; *p* = 0.068). The HS risk in subjects with MA (1.62; 95% CI 0.87–3.03; *p* = 0.129) was not significant. Compared with control subjects, the risk of HS was greater in females with any type of migraine (1.55; 95% CI 1.16–2.07; *p* = 0.003) and in female migraineurs under 45 (1.57; 95% CI 1.10–2.24; *p* = 0.012) (Table 1).

Lastly, a case–control study reported that migraine is an independent risk factor for aneurismal rupture (OR = 2.4; CI 95% 1.1–5.1), even if, as headache a premonitory symptom of aneurismal rupture, recall biases might have affected the study results (24).

## Headache as a Symptom Associated with IS

The relationship between migraine and stroke is bi-directional (25). Headache is associated with IS in 17–34% of patients (26) and bears its own classification in the 2013 IHS classification [headache attributed to IS (cerebral infarction) 6.1.1]. It is described as a new headache developing simultaneously with or in close temporal relationship to signs or other evidence of IS associated with neuroimaging confirmation of ischemic infarction. It is more frequently observed in posterior strokes (OR = 2.15 CI 95% 1.23–3.77; *p* = 0.0076). Medina et al. (27) reported that headache accompanied the ischemic event in 59% of patients with a transient ischemic attack (TIA) or stroke in the anterior circulation, and in 75% of patients with posterior ischemia. Headache is present at onset in 43–60% of cases, is persistent in 25–30%, and starts after in 14–27% of ischemic events. Headache associated with IS is more frequently observed in migraineurs (27), where it may sometimes mimic a migraine attack, although the studies reporting headache as a symptom of stroke are not recent and do not take into account the HIS classification for a definite diagnosis of the headache type.

## Migraine and Vascular Risk Factors

In the past, migraine has been associated with an unfavorable cardiovascular risk profile and a higher risk for coronary artery disease when stratified by the Framingham risk score (28). In a population-based study carried out in the Netherlands, the cardiovascular risk profile of 620 adult migraineurs was compared to that of non-migraineurs. The migraineurs were more likely to be smokers (OR = 1.43; 95% CI 1.1–1.8), to have a parental history of early myocardial infarction, and were less likely to be alcohol consumers (OR = 0.58 95% CI 0.5–0.7). Furthermore, MA patients tended to have high-blood pressure (OR = 1.76 95% CI 1.04–3.0), a worse cholesterol profile (total cholesterol ≥240 mg/dL: OR = 1.43 95% CI 0.97–2.1), and an earlier onset of coronary heart disease or stroke (OR = 3.96; 95% CI 1.1–14.3), with a twofold probability of having an elevated Framingham risk, resulting in a higher cardiovascular risk profile (29).

The recent cross-sectional, population-based HUNT study (30) assessed parameters such as blood pressure, body mass index (BMI), serum total, and high-density lipoprotein cholesterol in 44,098 subjects so as to calculate the Framingham 10-year risk score for coronary death and myocardial infarction (30). The authors reported an unfavorable cardiovascular risk profile, as established by an elevated Framingham risk score, both in migraine with (OR = 1.54; 95% CI 1.21–1.95) and without aura (OR = 1.17; 95% CI 1.04–1.32) and in non-migrainous headache (OR = 1.17; 95% CI 1.10–1.26). Interestingly, there was a consistent rise in the Framingham risk score in line with a higher headache frequency, suggesting that more frequent headaches and long-term migraine may worsen the cardio-metabolic profile in migraineurs, with a higher Framingham risk score and risk of developing atherosclerosis, insulin-resistance, and metabolic syndrome (31). The increased risk in the HUNT study on non-migrainous headache and MO was accounted for by lifestyle factors, such as low-physical activity, smoking, and a high BMI. Conversely, these factors did not completely explain the elevated risk in the MA patients, suggesting that other, peculiar mechanisms underlie the elevated risk in MA (30).

On the other hand, more recently, the association between migraine and vascular events has been demonstrated to be independent from the common vascular risk factors, above all in the young adult population with MA, although the smoking habit and use of oral contraceptives most surely increase the IS risk in female migraineurs (8, 32).

A multicentric Italian study (33) on 981 young adult IS patients under 45, 50.7% females, was carried out to evaluate whether the vascular risk profile variation had a predictive value on the migraine subtype. Medical history included the presence of headache attacks, any cardiovascular risk factors, the presence of a PFO, the V-Leiden factor, and prothrombin mutations (coagulative factor II mutation). The MA risk increased as the number of cardiovascular risk factors decreased (OR = 0.50; 95% CI 0.24–0.99 for 2 factors or more) and the number of thrombophilic variants increased (OR = 2.21; 95% CI 1.05–4.68 for carriers of at least 1 of the 2), or in the presence of right-to-left shunt (RLS) (OR = 2.41; 95% CI 1.37–3.45). None of these factors influenced the risk of MO. Therefore, there seems to be an inverse relationship between migraine and vascular risk factors in stroke at a young age. Indeed, young patients with IS had a low-cardiovascular risk profile and a pro-coagulative state, while the presence of a PFO was associated to MA. In the discussion section, the authors propose, on the basis of these data, that the small ischemic episodes caused by PFO microembolisms may well give rise to a cortical spreading depression (CSD) and migraine attacks and that these episodes may be favored by the presence of altered coagulation that increases both the small embolisms and those with a symptomatic clinical significance (33).

In agreement with these findings, another study demonstrated a 1.5 greater IS risk (95% CI 1.1–2.0) in women aged 15–49 years with probable MA (with visual aura), which was higher in those with no history of hypertension, diabetes, or myocardial infarction compared to women with no migraine. Women with probable MA who were current cigarette smokers and current users of oral contraceptives had sevenfold higher odds of IS (95% CI 1.3–22.8) than did women with MA who were non-smokers and did not use oral contraceptive (11).

From another perspective, the Italian multicentric study MIRACLE (34) was carried out on 2,973 patients with a diagnosis of hypertension or migraine in a general practitioner setting, where 570/2,973 had both pathologies. The group with both pathologies (where the onset of comorbidity occurred at about 45) had a later onset of migraine, compared to the migraine-only group, and an earlier onset of hypertension than the hypertension-only group. This comorbidity group also had a higher prevalence of cerebrovascular events than the group with only one factor: 4.4 vs. 0.7%, in the migraine-only group and 3.1% in the hypertension-only group. There was a fivefold prevalence of a history of stroke/TIA for the age group 40–49 in the comorbidity group, compared to the hypertension-only group for the same age range (4.8 vs. 0.9%) (34).

In summary, the current evidence suggests that, at a young age, MA is directly associated with IS, in the presence of fewer traditional cardiovascular risk factors which, conversely, seem to increase the risk for IS in older migraineurs, worsening the cardio-metabolic profile.

### Migraine and obesity

Obesity is another relevant cardiovascular risk factor to have been associated with migraine, especially with a higher frequency of pain crises, although the studies assessing such a relationship between migraine and obesity have shown conflicting results, which might well be due to methodological differences (self-reported BMI in some studies, direct measurement in others, mainly in those confirming the association).

Bigal et al. (35) showed that, although migraine prevalence was not associated with BMI, the proportion of subjects with severe headache pain increased as did the BMI, doubling in the morbidly obese group (BMI ≥35 kg/m^2^) compared to the group with a normal BMI (BMI 18.5–24.9 kg/m^2^). Indeed, a higher BMI was directly correlated to a high number of headache crises (35).

Moreover, another study (36) reported that the prevalence of “transformed” migraine ranged from 0.9% in normal weighted subjects to 1.2% in overweight subjects (OR = 1.4; 95% CI 1.1–1.8), 1.6% in obese subjects (OR = 1.7; 95% CI 1.2–2.4), and 2.5% in severely obese subjects (OR = 2.2; 95% CI 1.5–3.2), implying that obesity is likely to be a risk factor for “transformed” migraine.

Furthermore, for a BMI ≥35 kg/m^2^, an OR of daily migraine of 3.11 (95% CI 1.12–8.67) was detected in women, along with an increased risk of phonophobia and photophobia and decreased risk of a unilateral pain characteristic and migraine aura, suggesting that the association between BMI with migraine is limited to migraine frequency and specific migraine features (37).

## Migraine and Coronary Heart Disease

Although three population studies have supported the relationship between migraine, especially MA, and coronary heart disease, an increased risk of cardiac events in migraineurs was not confirmed by a meta-analysis.

The Women’s Health Study (38) demonstrated a more evident association between MA and both cardiovascular disease and stroke in 27,519 female patients (3,577 migraineurs) with an 11.9 year follow-up, stratified by the Framingham risk score for coronary heart disease, in the presence of a low-Framingham score. Female migraineurs with a low-Framingham score had an OR of 3.88 (95% CI 1.87–8.08) for IS and 1.29 for myocardial infarction (95% CI 0.40–4.21). The females with MA in the high-risk group had an OR of 1.0 (CI 95% 0.24–4.14) for stroke, and 3.34 (95% CI 1.50–7.46) for myocardial infarction. Women with MO did not have any increased risk of myocardial infarction or IS in any of the Framingham risk score groups (38).

In the Atherosclerosis Risk in Communities Study, patients with headache were roughly twice as likely to have a history of angina than were controls and the MA group had the highest risk score (39).

Furthermore, as part of the Physician’s Health Study, men with any migraine (with or without aura) were reported to have at increased risk for major cardiovascular disease (HR: 1.24; 95% CI 1.06–1.46), a finding supported by a 42% increased risk of myocardial infarction (40).

However, in the aforementioned meta-analysis by Schurks et al. (8), which confirmed the increased IS risk in migraine, there was neither statistically significant difference for the association between any migraine and myocardial infarction: OR = 1.12 (CI 95% 0.95–1.32) nor for the association between migraine and death due to cardiovascular events: OR = 1.03 (CI 95% 0.79–1.34).

Lastly, a population-based study found that after adjustments for gender, age, disability, treatment, and vascular risk factors, migraine remained significantly associated with myocardial infarction (OR = 2.2; 95% CI 1.7–2.8), stroke (OR = 1.5; 95% CI 1.2–2.1), and claudication (OR = 2.69; 95% CI 1.98–3.23) (41).

## Migraine and Patent Foramen Ovale

The association of migraine and the presence of PFO have attracted a great deal of attention and study over the last few years.

Patent foramen ovale has been observed in approximately 40–60% of people with MA (42) as compared to about 25% of the general population (43). A systematic review of literature, using meta-analysis to combine the results of different studies showed that the OR of PFO in patients with migraine compared with the OR of PFO in a group of patients without migraine was 2.54 (95% CI 2.01–3.08) (42).

Such an association between migraine and PFO is bi-directional, since subjects with PFO have a fivefold higher risk of suffering from migraine (44, 45). However, there are also data pointing against the correlation between PFO and MA. The only population-based study investigating the association between PFO and migraine with or without aura was cross-sectional and found no association (46). The limitations of this study are the use of transthoracic echocardiography to evidence the PFO (which has a low diagnostic accuracy in detecting atrial defects), the age group was older for migraine patients and self-reporting of disease may not be reliable (47).

A report documented that the occurrence of atrial shunts was consistent with autosomal-dominant inheritance in some families with MA (48). The presence of a PFO seems to run in families: siblings of stroke patients with PFO have a significantly higher prevalence of PFO than do those without, for brothers (OR = 3.64; *p* = 0.015) and for sisters (OR = 9.8 *p* < 0.01) (49). Subjects with PFO and migraine have a higher probability of having a family history of migraine than those who have migraine alone without PFO.

Some theoretical pathophysiological mechanisms may be involved in PFO leading to migraine. It has been observed that small particles and air bubbles in animals may trigger CSD and, therefore, there is a possibility that PFO may promote migraine attacks in some patients. Moreover, vasoactive amines and other compounds that are usually removed at a pulmonary level, like prostaglandins, serotonin (pulmonary removal 85–95%), angiotensin I not converting into angiotensin II and bradykinin, may bypass the pulmonary filter through the PFO and trigger migraine (50). However, if RLS is involved in the pathogenesis of MA, then the degree of the RLS might be postulated to affect the clinical picture of patients with aura and migraine headache. Conversely, some data show that RLS does not seem to affect the clinical manifestation of MA and that the extent of RLS fails to correlate with the severity of the clinical picture of the disorder, against the migraine/PFO nexus (51, 52).

Patients with migraine and a history of IS have ample RLSs (53). The percutaneous closure of septal defects with a left-to-right shunt has been associated with improvement of migraine. Although non-randomized studies have documented that migraine attacks frequency decrease after percutaneous closure of the RLS (54), to date, the only randomized study, i.e., Migraine Intervention with STARFlex Technology, MIST, carried out on this topic, did not confirm these results, giving rise to substantial controversy (55).

However, there are several limitations that should be taken into consideration in these studies, including there being no control group, the possible impact of incomplete closure of the atrial shunt, the retrospective design, implicating recall bias, the placebo effect that may lead to up to 70% reduction of attacks frequencies, the potential prophylactic effect of aspirin administered after PFO closure.

Moreover, the risks involved in PFO closure must not be underestimated. Literature reports that up to 8% of transcatheter interventions may require surgical procedures to manage complications (56). Therefore, it may be concluded that, currently, PFO closure is not a recommended routine procedure to prevent migraine.

## Migraine and Cervical Artery Dissection

A recent meta-analysis (57) has demonstrated that migraineurs have a twofold risk of dissection of the cervical arteries (CAD) (OR = 2.06; CI 95% 1.33–3.19). In a hospital-based prospective case–control study, which assessed personal and family history of migraine in 72 patients with CAD, 72 patients with cerebral infarct unrelated to a CAD (non-CAD), and 72 control subjects, personal history of migraine was significantly associated to CAD compared to non-CAD (59.7 vs. 30.6%; OR = 3.14; 95% CI 1.41–7.01) and controls (18.1%; OR = 7.41; 95% CI 3.11–17.64). As opposed to MA, MO had a significantly higher frequency among those with CAD than among those without (56.9 vs. 25.0%; OR = 3.91; 95% CI 1.71–8.90) and controls (58).

One case–control study (59) on 313 patients with CAD vs. controls clearly demonstrated that migraine was more frequently observed in subjects with CAD than in controls (36 vs. 23% OR = 2.15; 95% CI 1.48–3.14) and that there was a higher association for MA patients (23 vs. 12%, OR = 2.41; 1.53–3.80), with females prevailing over males. Migraine was more common among patients with CAD and stroke, than in stroke patients without CAD (35.7 vs. 27.4%, *p* = 0.003) (60), in agreement with the hypothesis that “migraine may represent a predisposing condition for cervical artery dissection” (58).

## Migraine and Genetic Pathologies

Migraine has been observed to be a prominent feature in the phenotype of several genetic vasculopathies and channelopathies (44). Familial hemiplegic migraine (FHM) is due to mutations in the CACNA1A gene, coding for the voltage-gated Ca_v_2.1 calcium channel α subunit (61). Transgenic knock-out mice with Ca_v_2.1 voltage-gated Ca^2+^channel mutations were demonstrated to be more prone to develop CSD (characterized by an intense depolarization of neuronal and glial membranes) and to have a higher susceptibility to IS with a faster than average depolarization at the artery occlusion and a quicker expansion of the infarction core at diffusion MRI. Therefore, these mice showed more extensive ischemic lesions and higher disabilities than did the control mice (62).

Migraine with aura is the earliest manifestation of “cerebral autosomal-dominant arteriopathy with subcortical infarcts and leukoencephalopathy” (CADASIL), and is observed in about 30% of cases. CADASIL, a disease belonging to a family of disorders known as “leukodystrophies,” is a pathology caused by mutations of the *Notch 3* gene on chromosome 19, which codifies a transmembrane receptor, expressed almost exclusively by nervous and vascular smooth muscle cells. There is a progressive degeneration of the smooth muscle cells in the blood vessels due to the accumulation of the Notch3 protein, observed as granular osmiophillic deposits at electron microscopy. The resulting damage may increase the susceptibility to CSD (63). Migraine has been observed in 44% of females with CADASIL and in 31% of males. While there is a male preponderance for stroke: 74 vs. 57% for females (64).

Susceptibility toward migraine is not only a feature of CADASIL but is also present in retinal vasculopathy with cerebral leukodystrophy (RVCL), in hereditary endotheliopathy with retinopathy, nephropathy, and stroke (HERNS) as well as in hereditary infantile hemiparesis retinal arteriolar tortuosity and leukoencephalopathy (HIHRATL) (65). RVCL is a neuro-vascular syndrome, caused by a mutation in the TREX1 gene, which starts with vision loss, followed by cognitive disturbances, depression, and migraine. An MRI investigation detected WMLs in the advanced stage. HIHRATL is due to a mutation in the COL4a1 gene, encoding the α1 chain of type 4 collagen. In the presence of this vasculopathy, the cerebral vessels usually show a destruction of the basal membrane, and enlargement of the endothelial cells, although the pathophysiological mechanisms linking these genetic vasculopathies to migraine are still unknown. However, common genetic susceptibility, enhanced susceptibility to CSD, and vascular endothelial dysfunction have been hypothesized to play a role (66, 67).

Lastly, another disease presenting stroke and migraine is the syndrome of mitochondrial myopathy, encephalopathy, lactic acidosis, and stroke (MELAS), caused by mutations in the mitochondrial DNA (68). Patients affected by MELAS usually present prolonged MA attacks, indicating that some kind of energy dysfunction may underlie migraine.

## Migraine and Autoimmune Pathologies

Headache is the main symptom in giant cell arteritis or Horton’s disease, but migraine, stroke, and loss of memory are frequent symptoms in antiphospholipid syndrome or antiphospholipid antibody syndrome, also known as Hughes syndrome (69, 70). Conversely, recent data show that migraineurs have a significantly higher prevalence of antiphospholipid antibodies, suggesting that the presence of comorbidity between the two conditions (71).

Migraine is observed in 52% of cases of Sjögren’s syndrome. Late onset of “migraine-like” episodes with prolonged sensori-motor deficits and concomitant neuropsychiatric disease are typical manifestations (72).

Migraine and headache of difficult classification are frequent findings in systemic lupus erythematous (SLE), along with multiple ischemic cerebral lesions (73). The question arises whether a specific “lupus headache” exists as a separate entity. Such an issue was addressed by Mitsikostas et al.’s study (74) of 2004. It pooled data from 8/35 studies (controlled and uncontrolled) identified in a literature search that used the IHS criteria and observed a 57.1% prevalence of headache of any type (migraine 31.7% and tension-type headache 23.5%) in SLE patients. Pooled data from seven controlled studies showed that the prevalence of all headache types, including migraine, did not differ from controls. No clear evidence was found for the concept of a “lupus headache” and neither specific pathogenetic mechanisms of headache in adult SLE patients were identified, nor was any association between headache and the disease status observed. Moreover, there was insufficient evidence that headache is associated with anxiety and depression in SLE (74).

Headache is the most common manifestation in primitive vasculitis of the central nervous system (75, 76), where it is mostly reported as a generic symptom.

## Pathophysiological Remarks

The following possible explanations have been proposed for comorbidity, i.e., the greater than coincidental association between migraine and other pathological conditions, like IS, by Mannix (77), Lipton and Silberstein (78):

1. One condition may cause the other.
2. There may be a common mechanism underlying both conditions.
3. Genetic factors may cause a susceptibility to two or more neurologic disorders.
4. Environmental factors may alter brain function in such a way so that the likelihood of the occurrence of two neurological disorders is increased.

Although rarely, an MA attack may directly cause an ischemic event, the “migrainous infarction,” as in the first scenario. Conversely, headache is a symptom associated with IS in 17–34% of patients (26) and bears its own classification in the IHS classification 2013 (coded as 1.4.3). Although migrainous infarction accounts only for a minority of migraine-related strokes, it does represent an useful model to highlight some pathophysiological linking migraine to IS. Indeed, migraine may be considered a “neuro-vascular” disease, where the vascular consequences of CSD (oligoemia and consequent increased intraparenchimal vascular resistance) may combine with a coagulopathy (79). A prolonged decrease in cerebral blood flow and neuronally mediated vasodilatation cause sluggish flow in large intracranial vessels during the aura migraine. Moreover, intravascular thrombosis could be favored by neurogenically mediated inflammatory responses caused by the release of vasoactive peptides, including NO, activation of cytokines, and upregulation of adhesion molecules (80).

However, most IS in migraineurs occur in the interictal phase, suggesting that an indirect relationship between migraine and IS (79), where the other three types of explanations of the comorbidity, according to Mannix, Lipton and Silberstein (77, 78) could play a role.

Mainly, migraine and stroke may be related to each other by shared pathophysiological mechanisms.

Indeed, endothelial dysfunction may be the pivotal “missing link” between migraine and vascular disease (79). Indeed, some studies have found that migraineurs have a lower endothelium-dependent vasodilatation capacity than do to non-migraineurs (81) due to a decreased NO bioavailability and an increased oxidative stress in migraine. Therefore, even from this perspective, migraine may be regarded as a “vascular disorder.” However, although endothelial dysfunction may be considered the final common pathway of the traditional vascular risk factors, including obesity, it is more likely to be directly related to the migraine pathophysiology, on the basis of the evidence previously reported that the association between migraine and vascular events seems to be independent from the common vascular risk factors, at least at young age (33).

As aforementioned, migraine increases the risk for CAD, which is involved in a small proportion of strokes and mainly in young subject. Indeed, some migraine patients have elevated serum elastase levels, which may indicate abnormalities of the vessel walls and predispose to dissection; conversely, some reports have described CADs that trigger migraine attacks (57, 82, 83).

Patent foramen ovale has been supposed to one of the biological mechanisms linking migraine to stroke. However, this migraine/PFO relationship might represent the result of a common genetic disposition leading, on the one hand, to migraine and, on the other, to endocardiac alteration with persistence of the foramen ovale (84) and, putatively, to artery wall laxity with propensity to spontaneous artery dissection. Hence, genetic factors could confer a susceptibility to migraine and stroke, as foreseen in the third scenario, according to Mannix (77). Indeed, as previously reported, migraine and stroke are prominent clinical features of the phenotype of several genetic vasculopathies (CADASIL, HRNS, HIHRATL, RVCL), channelopathies (FHM), and mitochondrial myopathies (MELAS). Although these are rare disorders and cannot justify most cases of migraine and stroke, they do, however, suggest that putative, still unknown genetic factors involved in the structure and function of cortical-meningeal vessels and in neuronal excitability regulation lie at the basis of such comorbidity. Moreover, since anatomical variants of the circle of Willis have been detected more frequently in migraineurs than in controls and, particularly, posterior anomalies in MA, a “vascular mechanism” has been hypothesized where a dysregulation in cerebral blood flow in regions supplied by the variant circle could lead to CSD (85).

As to the role of environmental factors in the relationship between migraine and stroke, migraine-specific drugs, e.g., ergot-derivatives and triptans, have been questioned, due to the possibility of their having adverse effects on the cerebral vasculature. However, data on ergotamine are controversial. A case–control study reported an increased risk of ischemic events in ergotamine abusers (86), especially in those on other cardiovascular treatments, while therapeutic dosages do not seem to affect the vascular risk. No significant association has been found for the use of these triptans in migraineurs and stroke incidence (87, 88).

Lastly, even acquired and genetic thrombophilic disorders have been hypothesized to contribute to the migraine–stroke comorbidity. Debate is still ongoing as to hyperhomocysteinemia, where some studies report increased homocysteine levels in migraineurs, particularly in MA, and an association with the C677 polymorphysms of the methylenetetrahydrofolate reductase mutations (MTHFR) gene (89). Moreover, there is a high incidence of thrombophilia in individuals with MA and PFO, in particular, in those with MTHFR, protein C, and protein S deficiency and the presence of thrombophilia increases the indication for closure (53, 90). However, further studies are needed to clarify this issue.

### Migraine, hemorrhagic stroke, and stress

Several hypotheses have been formulated as to the mechanisms that link hemorrhage to migraine and include the presence, in migraineurs, of a vessel wall pathology (a dysfunction in the endothelial cells, an increase in the vessel resistance), a platelet dysfunction, moreover, the presence of arteriovenous malformations, though these are rare causes of migraine (57, 82, 83).

**Figure 1:**
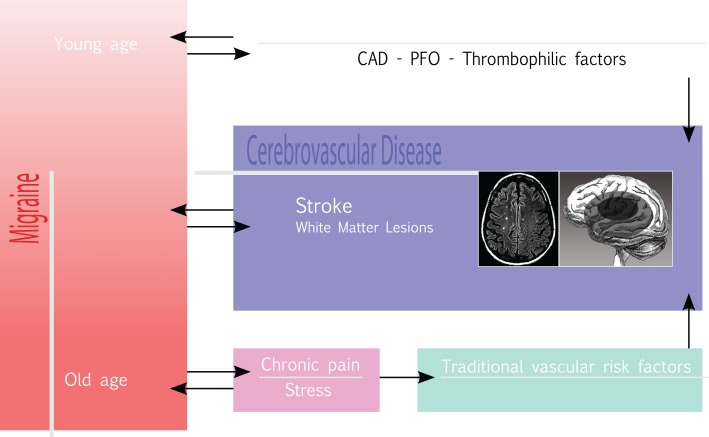
**The migraine-stroke interplay: pathophysiological connections**.

Based on some initial evidence as to an association between stroke and stress on the one hand, and on the well-known, complex relationship between migraine and stress (91), on the other, even stress may play a role in increasing the risk for HS in migraine. The case–control study INTERSTROKE (92), carried out in 22 countries on a total of 3,000 stroke cases (78%, with IS; 22%, with intracerebral HS), and 3,000 controls, demonstrated a statistically significant association between ictus and psychosocial stress (OR = 1.30 95% CI 1.06–1.60) and depression (OR = 1.35, 95% CI 1.10–1.66). Also, the longitudinal population-based study, Chicago Health and Aging Project (93), carried out in Chicago with individual interviews on a population of African Americans and non-Hispanic white subjects aged ≥65, showed a statistically significant association, corrected for risk factors, between stress and stroke mortality. This was confirmed, at secondary analysis for stroke subtypes, for HS (OR = 1.70; 95% CI 1.28–2.25). Furthermore, two case–control studies (94, 95) have reported that even psycho-physical stress and self-perceived psychological stress are associated to an increased risk of IS, supporting a possible role for stress as a risk factor for stroke. Hence, in our hypothesis, stress consequent to pain in migraine, especially when chronic, may represent a “missing link” in the complex interplay between migraine and stroke (Figure 1), where a higher headache frequency and long-term migraine may worsen the cardio-metabolic profile in migraineurs, with a higher risk of developing atherosclerosis, insulin-resistance, and metabolic syndrome (31). However, further studies are required to support this hypothesis.

## Clinical Management Implications

Migraine patients are followed-up in headache centers that mainly aim at pain attack prevention, while less attention is usually paid to cardiovascular prevention. Conversely, the comorbidity between migraine and stroke should be carefully considered in the clinical management of migraine patients, mainly for treatment regimes. As MA is now recognized as a well-defined risk factor for IS, patients given an MA diagnosis should be screened for other, concomitant vascular risk factors, as recently suggested by Sacco et al. (96), especially if not young and/or in the presence of WMLs at neuroimaging. However, migraineurs with a normal neurologic examination do not require routine MRI. Modifiable vascular risk factors should be carefully treated, in migraineurs, with proper pharmacological and, if need be, behavioral strategies, to reduce the global burden of the patient’s vascular risk. As good clinical practice suggests in all cases of comorbidities, it is advisable to choose drugs that are potentially useful for both migraine prophylaxis and the treatment of the associated conditions affecting the vascular risk. For example, in the case of hypertension, the use of angiotensin-converting enzyme inhibitors, may be a reasonable choice, considering that such drugs have yielded some preliminary evidence, on the one hand, of a reduction of the risk of vascular events in normotensive subjects, and on the other, have been of benefit in migraine prophylaxis (96). However, the American Heart Association/American Stroke Association 2011 guidelines (97) for the primary prevention of the IS state that “*because there is an association between migraine frequency and stroke risk, treatments to reduce migraine frequency might be reasonable, although there are no data showing that this approach would reduce the risk of a first stroke*.” Lastly, the use of oral contraceptives should be discouraged in all female patients suffering from MA and, in subjects with MO, in the presence of concomitant vascular risk factors, thrombophilic conditions (96), and/or being over 35.

## Conclusion

Migraine, mostly MA and IS are interconnected by a complex bi-directional relationship. Migraineurs have about a twofold higher risk of IS and although this risk increase has been well-defined for MA, this does not apply to MO. The risk is further increased by female gender, being under 45, smoking, and/or being on oral contraceptive. There is also an increased independent risk of clinically silent WMLs for female migraineurs.

Migraine is also associated with increased risk of HS, mostly in young women.

Although the interplay between migraine, IS, and cardiovascular risk factors is manifold and controversial, it is likely that migraine is directly associated with IS at a young age, where there seems to be an inverse relationship between MA and the traditional vascular risk factors. Conversely, in older migraineurs, the cardiovascular risk factors may play a role in linking migraine to stroke, based on the finding that long-term, chronic migraine seems to worsen the cardio-metabolic profile in migraineurs by mechanisms, which might include chronic stress induced by pain, even if this hypothesis deserves confirmation.

The relationship between migraine and stroke could also involve PFO, CAD, and genetic vascular pathologies, whereas such conditions may share with migraine common genetic predisposing traits. Although the prevalence of PFO has been proven to be significantly higher in patients with MA than in healthy controls, the relationship between migraine and PFO remains controversial and PFO closure is not a recommended procedure to prevent migraine.

Lastly, although the pathophysiological mechanisms underlying the comorbidity between migraine and IS remain to be fully elucidated, the evidence linking MA to IS is such that patients with MA should be screened for other concomitant conventional vascular risk factors and treated accordingly.

## Conflict of Interest Statement

The authors declare that the research was conducted in the absence of any commercial or financial relationships that could be construed as a potential conflict of interest.
